# Motor training is improved by concurrent application of slow oscillating transcranial alternating current stimulation to motor cortex

**DOI:** 10.1186/s12868-022-00731-x

**Published:** 2022-07-15

**Authors:** Martin V. Sale, Anastasiia Kuzovina

**Affiliations:** School of Health and Rehabilitation Sciences, The University of Queensland, St Lucia, Brisbane, QLD 4072 Australia

**Keywords:** tACS, Transcranial alternating current stimulation, Motor training, Encoding, Slow-wave oscillations, Neuroplasticity, Non-invasive brain stimulation

## Abstract

Physical exercise and neurorehabilitation involve repetitive training that can induce changes in motor performance arising from neuroplasticity. Retention of these motor changes occurs via an encoding process, during which rapid neuroplastic changes occur in response to training. Previous studies show that transcranial alternating current stimulation (tACS), a form of non-invasive brain stimulation, can enhance encoding of a cognitive learning task during wakefulness. However, the effect of tACS on *motor* processes in the awake brain is unknown. In this study, forty-two healthy 18–35 year old participants received either 0.75 Hz (active) tACS (or sham stimulation) for 30 min during a ballistic thumb abduction motor training task. Training-related behavioural effects were quantified by assessing changes in thumb abduction acceleration, and neuroplastic changes were quantified by measuring motor evoked potential (MEP) amplitude of the *abductor pollicis brevis* muscle. These measures were reassessed immediately after the motor training task to quantify short-term changes, and then 24 h later to assess longer-term changes. Thumb abduction acceleration in both active and sham stimulation conditions increased immediately after the motor learning, consistent with effective training. Critically, participants in the active group maintained significantly higher thumb acceleration 24 h later (t_40_ = 2.810, P = 0.044). There were no significant changes or inter-group differences in MEPs for both conditions. The results suggest that 0.75 Hz tACS applied during motor training enhances the effectiveness of motor training, which manifests as enhancement in longer-term task benefits.

## Introduction

Neuroplasticity describes the process of cortical reorganisation in response to internal and external stimuli, and is important for processes such as memory and learning [1–3]. Learning consists of two stages: encoding, which involves temporary rapid neuroplastic changes in synaptic strength, and consolidation, which involves more permanent changes [4]. Motor learning is a type of procedural memory encoded through repeated task practice [5].

It is widely accepted that the non-rapid eye movement (NREM) phase of sleep, characterised by endogenous slow-wave (0.5-4 Hz) oscillations in neural activity, is involved in the consolidation of spatial, declarative and procedural memory [6, 7]. Conversely, sleep deprivation has detrimental effects on cognitive function, attention, long-term and working memory [8].

Given the important role of slow-wave oscillations in promoting learning, there has been considerable research interest in promoting slow-wave oscillatory activity within the brain. One possible approach is to harness the beneficial aspects of slow wave sleep by entraining these oscillations “artificially” using brain stimulation techniques. For example, a form of non-invasive brain stimulation (transcranial alternating current stimulation; tACS) has been used to entrain physiologically-relevant neural oscillations, with subsequent effects on task performance [9, 10]. This form of brain stimulation differs from other forms of non-invasive brain stimulation, such as repetitive transcranial magnetic stimulation (rTMS) and transcranial direct current stimulation (tDCS). Unlike these other forms of brain stimulation, which cause longer-term changes in cortical excitability via effects on plasticity mechanisms (for review see [11, 12]), tACS affects neural oscillations. Although the physiological mechanisms underlying tACS are not well understood, emerging research in non-human primates has shown that tACS can affect the timing, but not the firing rate of neurons [13]. tACS has been applied at various frequencies, with resultant effects on associated tasks. For example, high frequency (40 Hz) gamma oscillations have been shown to improve perceptual learning of a phoneme categorization task [14]. Similarly, application of alpha (10 Hz) tACS to parieto-occipital cortex improved target detection performance [15]. Previous studies have also demonstrated efficacy for slow oscillating tACS in enhancing the effects of sleep-like processes. For example, slow oscillating (0.75 Hz) tACS applied during sleep improves the recall of declarative memories in healthy populations [16]. Using a similar rationale, fast spindle activity has been targeted with tACS during sleep, and improvements in motor memory consolidation have been reported [17].

In a major conceptual advance, the cortical oscillations beneficial during sleep were shown to also influence brain function during *wakefulness*, thereby arguing for a direct causal role of neural oscillations in brain function (i.e., not just an epiphenomenon associated with sleep). Kirov et al. [9] investigated the application of slow oscillatory (0.75 Hz) tACS on the awake brain during a declarative memory task. When tACS was applied during the task (i.e., to enhance encoding), a significant improvement in immediate recall was observed following the completion of the task. The Kirov et al. [9] study investigated slow oscillatory tACS applied to the frontal cortex – whether the effects generalise to other cortical regions (e.g., the motor system) has been only studied in one previous research study [18]. Furthermore, the subsequent long-term effect of the stimulation on recall was not investigated, therefore it is uncertain whether slow oscillatory tACS has any longer-term effects on plasticity. This is an important element to explore, as other studies have supported the existence of cortical consolidating mechanisms susceptible to non-invasive brain stimulation when the stimulation is applied during learning tasks [19].

The present study aimed to investigate whether the application of slow wave 0.75 Hz tACS during a motor learning task (ballistic thumb abduction) assists in the encoding and subsequent consolidation of the task. The motor training task has been shown previously to increase motor cortical excitability and motor performance [20]. Changes in cortical excitability were quantified by measuring the amplitude of transcranial magnetic stimulation (TMS)-induced motor evoked potentials from a hand muscle in the trained hand, and motor performance was quantified by measuring thumb acceleration. TMS has been used extensively to quantify training-related changes (e.g., [20, 21]) and also to probe changes in cortical excitability induced with tACS [18, 22], and thus is a well-established and appropriate measure to quantify changes associated with motor training and tACS. It was hypothesised that participants receiving active tACS would show greater immediate changes (in cortical excitability and motor performance), and greater retention of the task 24 h later, compared with sham stimulation.

## Materials and methods

### Participants

Forty-two healthy participants were involved in the study (20 females, range 18–30 years, mean 23.1 ± 2.1 years) which was approved by the University of Queensland Human Research Ethics Committee. Participants were randomly assigned into either an active stimulation group (n = 21, 10 females, mean age 22.5 ± 1.9, LQ 0.91 ± 0.16) or a control group, which received sham stimulation (n = 21, 10 females, mean age 23.7 ± 2.3, LQ 0.90 ± 0.15). All participants provided written informed consent prior to participating in the study. Each participant completed a TMS safety screening questionnaire [23] and the Edinburgh Handedness Inventory [24] which provides a laterality quotient as a measure of hand dominance. The inclusion criteria involved right-hand dominant males and females aged 18–35 years old. Participants with neurological conditions, contraindications to TMS/tACS or traumatic injury to their left hand and/or thumb were excluded prior to the commencement of the study.

### Study design

The study undertaken was a quantitative single-blind randomised control study. Two main dependent variables were assessed during the study: acceleration of left thumb abduction and the motor evoked potential (MEP) peak-to-peak amplitudes of the right motor cortical representation of the left *abductor pollicis brevis* (APB) muscle. Participants were required to attend two experimental sessions. The first session involved the testing of the dependent variables before and immediately after the motor training task. The second session followed 24 h after the completion of the motor training task, and involved reassessment of the dependent variables, and allowed for investigation of longer-term, consolidatory changes in neuroplasticity.

### Experimental procedure

Surface electromyography (EMG) recordings of the left *abductor pollicis brevis* (APB) muscle were obtained using bipolar surface electrodes, placed in a belly-tendon configuration. A ground electrode was placed on the anterior forearm approximately 5 cm proximal to the left wrist. EMG signals were amplified 1000 times, filtered at 20-1000 Hz, digitised at 2 kHz via a CED 1401 interface (Cambridge Electronic Design, Cambridge UK) and recorded using Signal software (Cambridge Electronic Design, Cambridge UK). All recordings were saved onto a computer for offline post-experimental analysis.

### TMS & MEP recording

Participants were seated comfortably in a chair facing a wall whilst their forearms rested on the table. Monophasic TMS (Magstim, Dyfed UK) was applied using a hand-held 70 mm figure-of-eight coil over the optimal position to consistently evoke the largest motor evoked potential (MEP) of the left APB muscle at a slightly suprathreshold stimulus intensity. The TMS coil was held tangentially over the scalp, with the handle pointing 45 degrees posterolaterally from the midline. When the ideal position was found, the coil position was marked with a pen to allow accurate and reliable stimulation of the optimal cortical position throughout the experiment. The TMS intensity was then adjusted so that it evoked a MEP of approximately 1 mV peak-to-peak amplitude. The intensity of the TMS was recorded for each participant to ensure the same intensity was used for subsequent re-assessments.

Participants were advised to remain as relaxed as possible during the TMS. Participants received 20 TMS pulses at 5 s intervals to the ideal position of the M1, whilst the peak-to-peak APB MEPs were recorded. The MEP acquisition was undertaken three times: before motor training (pre-training), 5 min following the completion of the motor learning task (5 min post-training), and once again in the experimental session 24 h later (24 h post-training). Trials in which there was pre-stimulus EMG activity were excluded from analysis (< 2% of trials were excluded).

### Recording of thumb abduction acceleration

Following MEP recording, participants remained comfortably positioned in a chair, whilst their left forearm was placed in a semi-pronated position with their posterior forearm resting against a wooden block. Two velcro straps were used to secure their arm in place. One strap was placed approximately 10 cm proximal to the wrist joint line, and the other strap was placed immediately distal to the PIP joints of their left 4 digits. The velcro straps secured the hand and forearm in place, to reduce the impact of movement overflow from other joints on the acceleration data. An ADXL326 triplanar accelerometer (Analog Devices) was secured to the posterior aspect of the participant’s distal phalanx of the left thumb using surgical tape. Participants were shown the required motion of thumb abduction by the experimenter, and were instructed to accelerate their thumb as quickly as possible in the horizontal plane. Participants briefly practised the movement (~ 5 repetitions) until the desired movement was achieved. A digital metronome was set to 30 bpm to cue participants when to perform the thumb abduction movement, and they were advised to rapidly abduct their thumb immediately following each metronome beat. Visual feedback was provided to participants via an online display of their peak-to-peak acceleration performance. Furthermore, participants also received strong verbal encouragement to engage in the task throughout their training to maximise training-related effects. Twenty thumb abductions were recorded prior to the commencement of the motor training task, immediately following the motor training task and then 24 h later. Participants were instructed to abduct their left thumb at maximal acceleration at a frequency of 0.5 Hz for 20 repetitions (e.g., one repetition every 2 s).

### Motor training (MT)

The motor training task consisted of 2 blocks of 15 min, and involved participants maximally abducting their left thumb at a frequency of 0.5 Hz (e.g., 900 abduction movements in total). The participants were cued to initiate each movement with the aid of a metronome. Participants were regularly verbally encouraged to perform at their best effort.

Following the first 15 min block, participants rested quietly for one minute, during which no tACS was delivered. At the completion of the second 15 min motor training block, the delivery of tACS was ceased.

### Slow oscillatory tACS

A Neuroconn DC-Stimulator (neuroCare Munich) was used to deliver 0.75 Hz tACS via two 4 × 4 cm electrode pads at a 1 mA intensity. One electrode was positioned above the left supraorbital ridge. The second electrode was positioned centrally over the marked M1 area on the scalp, corresponding with the *APB* region identified during the MEP recording. In both active and sham conditions, stimulation was ramped up over 8 cycles at the beginning of the stimulation period, and ramped down over 8 cycles at the end of the period. Although we did not specifically assess participants’ perceptions of the stimulation conditions, several studies have previously reported that participants are unable to distinguish between conditions when ramps are applied at the start and end of stimulation with the stimulus intensity used in the present study [25–28]. Further, all participants had never previously received TMS or tACS, and were told that irrespective of the stimulation condition, they may or may not perceive the stimulation, thus we don’t consider detection bias a significant issue in interpreting the results. Participants in the active stimulation group received 2 blocks of 15 min stimulation during the motor learning task (one for each motor training block). Participants in the sham group received the stimulation for 2 blocks of 30 s only (one for each motor training block).

### Statistical analysis

Participant characteristics (age, handedness, TMS intensities, and baseline measures of MEP amplitude and thumb acceleration) were analysed through a series of two-tailed t-tests to compare the active and sham stimulation groups. Mean peak-to-peak MEP amplitude and thumb acceleration was calculated for each participant at each time point (baseline, 5 min post-MT, 24 h post-MT). For MEP amplitude and thumb acceleration, a separate two-way ANOVA with within-subject factor TIME (baseline, 5 min post-MT, 24 h post-MT) and between-subject factor CONDITION (active, sham) was conducted. In all cases, significant effects were followed up with two-tailed t-tests to compare measures between the active and sham groups at each time point of the experiment in both MEP and thumb abduction acceleration outcome measures, and the Bonferonni correction for multiple comparisons was used (the correction has been applied to the reported p value, so that p < 0.05 indicates significance). In cases of nonsphericity, the Huynh–Feldt correction was used. Data analysis was carried out with JASP (v0.16.2; JASP Team).

## Results

### Participant characteristics

Data from 42 participants were included in the results analysis, and no adverse effects were noted. There were no significant differences in the mean age (t_39_ = 0.246, P = 0.807) or handedness (t_39_ = 0.387, P = 0.471) between the active and sham groups. There were no significant differences in mean TMS intensities used to obtain baseline (pre-training) MEPs (mean sham intensity = 68.5% stimulator output; mean active intensity = 69.3% stimulator output; t_39_ = 0.523, P = 0.606). Baseline mean thumb abduction acceleration was comparable between the two groups (t_39_ = 0.390, P = 1.000), as was baseline mean MEP amplitude of the left APB (t_39_ = 0.143, P = 0.888). Therefore, any changes arising from tACS are unlikely to be attributed to differences in baseline participant characteristics.

### Thumb abduction acceleration

**Fig. 1:**
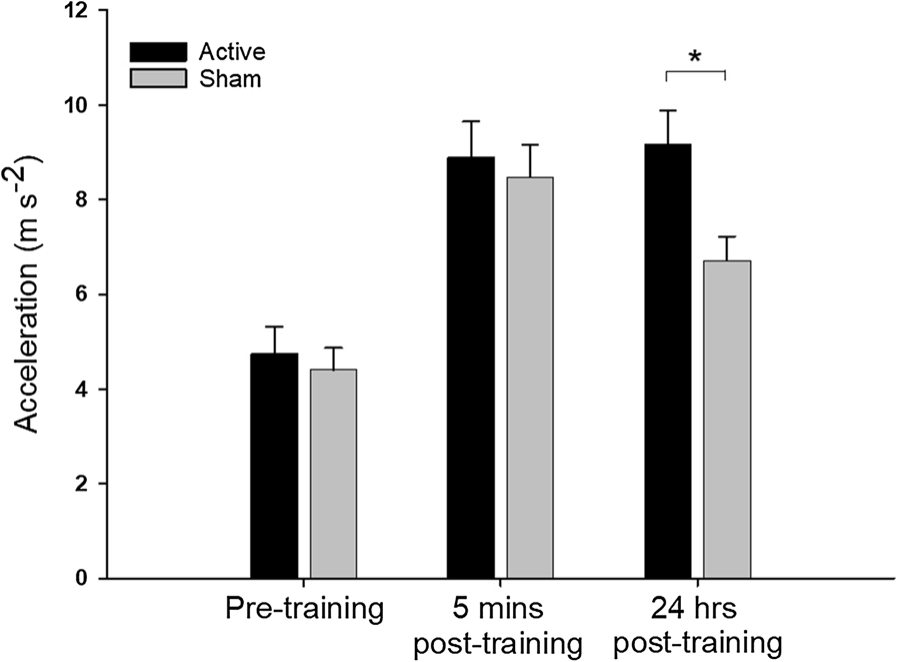
Mean (± SEM) acceleration data (n = 28) for participants performing 30 min of ballistic motor training with concurrent active 0.75 Hz tACS (black bars) or sham tACS (grey bars). Acceleration measures were obtained prior to training (pre-training), 5 min after training and then 24 h after training. Training increased acceleration across both groups at the 5 min post-training time point. Acceleration 24 h after training was greater than baseline in both groups, however, acceleration in the active tACS was greater than sham stimulation at this time point (*)

The mean horizontal thumb abduction results for the two groups, at each time point is shown in Fig. 1. ANOVA revealed no significant effect of condition (F_(1,40)_ = 2.216, P = 0.144, η_p_^2^ = 0.053) but there was a significant effect of time (F_(2,80)_ = 51.155, P < 0.001, η_p_^2^ = 0.561, ε = 0.927). Five minutes following the completion of the motor training task, a significant increase in mean thumb abduction acceleration was observed in both the active (t_19_ = 6.766 P < 0.001) and sham (t_19_ = 6.649, P < 0.001) groups compared to the respective baseline measures. This is consistent with previous research showing repeated abduction increases acceleration (see Sale et al., 2013). ANOVA also revealed a significant condition x time interaction (F_(2,80)_ = 3.882, P = 0.028, η_p_^2^ = 0.089, ε = 0.927), indicating that the change in acceleration across time was not consistent between groups. There was no significant difference between the two groups 5 min following training (t_39_ = 0.471, P = 1.000). Critically, there was a significant difference in acceleration between the two groups 24 h following the motor learning task (t_39_ = 2.810, P = 0.044) in which the mean thumb abduction acceleration of the active stimulation group was higher than the sham group. In both the active and sham groups, the mean thumb abduction acceleration 24 h following the motor learning task remained significantly higher than the baseline measure (active t_39_ = 7.239 P < 0.001, sham t_39_ = 3.770, P = 0.005), though not significantly different to the measure 5 min following the motor learning task (active t_39_ = 0.473 P = 1.000, sham t_39_ = 2.88, P = 0.077). These results suggest that participants who received tACS during the motor training showed greater improvements in thumb abduction acceleration 24 h following the motor training task compared to participants who received the sham stimulation.

### Motor evoked potential amplitude

**Fig. 2:**
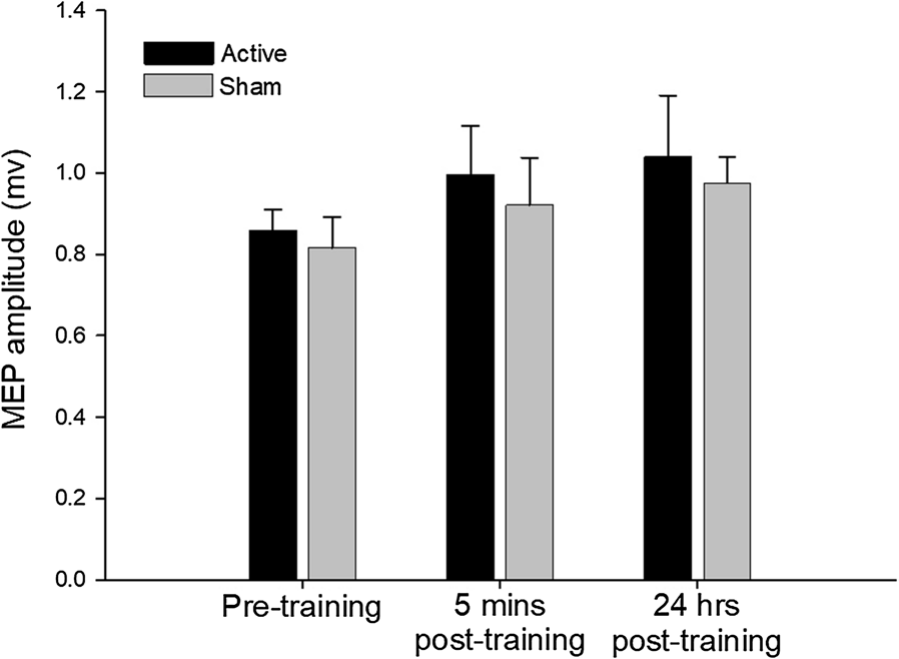
Mean (± SEM) MEP data (n = 28) for participants performing 30 min of ballistic motor training with concurrent active 0.75 Hz tACS (black bars) or sham tACS (grey bars). MEP measures were obtained prior to training (pre-training), 5 min after training and then 24 h after training. There was no effect of training or tACS on MEP amplitude

The mean MEP amplitudes of the left APB for the two groups at each time point is shown in Fig. 2. ANOVA revealed no reliable effect of condition (F_(1,40)_ = 0.469, P = 0.498, η_p_^2^ = 0.012) nor reliable effect of time (F_(2,80)_ = 1.564, P = 0.215, η_p_^2^ = 0.038, ε = 1.000), nor a significant condition x time interaction (F_(2,80)_ = 0.013, P = 0.988, η_p_^2^ < 0.001, ε = 1.000). Five minutes following the motor training task, there was no significant change in mean MEP amplitudes for both the active and sham groups, compared to baseline (active t_39_ = 0.973 P = 1.000; sham t_39_ = 0.784 P = 1.000). There was no significant difference between groups at this time point (t_39_ = 0.519, P = 1.000). MEP amplitudes obtained 24 h after training were not significantly different compared to the measurements taken 5 min post-MT (active t_39_ = 0.320; P = 1.000; sham t_39_ = 0.391 P = 1.000). The mean MEP amplitudes 24 h after training were not significantly different, compared to the baseline measure in both groups (active t_39_ = 1.293 P = 1.000; sham t_39_ = 1.140 P = 1.000). There were no significant differences in the active and sham group 24 h following MT (t_39_ = 0.451, P = 1.000). These results suggest that there is no significant effect of tACS, or the motor training task, or an interaction between tACS and concurrent motor training, on the excitability of the right cortical representation of the left APB.

## Discussion

The repetitive performance of motor tasks leads to the acquisition of training-related changes [29]. Human learning relies on the process of encoding to rapidly acquire new skills [9], and consolidation in order to retain neuroplastic changes in the long-term [30], resulting in more permanent skill acquisition. Enhancing encoding of skills can therefore facilitate more effective learning. Previous studies have supported the efficacy of tACS in the encoding of *declarative* memories in healthy participants [9], however, there is no research into tACS for the encoding of procedural and motor memory. In the present study, we investigated whether tACS over the motor cortical representation activated during a ballistic thumb abduction motor training task enhanced the encoding or consolidation of the task. We show that slow oscillatory tACS applied during motor training can enhance performance benefits 24 h after training compared to sham tACS. That is, although both active and sham stimulation groups show improvements in thumb acceleration 24 h after training, the group that received active tACS show a greater improvement.

The use of non-invasive brain stimulation, including tACS, has been seen as a promising adjunct approach for promoting training- and learning-related changes. A novel approach has been to apply tACS during wakefulness at a slow oscillatory frequency to mimic effects of sleep [9], which is known to influence neuroplasticity [31]. Here, declarative memory was improved if slow oscillatory (0.75 Hz) tACS was applied during the learning task. By applying tACS during the task, processes associated with encoding were targeted. The slow oscillatory tACS led to an increase in not only delta but also theta power in the electroencephalogram. The authors speculated that the theta increase was important in promoting the encoding of the learnt information [9]. In the present study we sought to apply a similar approach but to target motor regions and motor processes rather than cognitive processes.

The results of the present study suggest that tACS applied at a slow oscillatory frequency can improve longer-term retention of motor training-related effects. As mentioned previously, the study was motivated by an earlier study using a similar approach in the cognitive domain [9]. In that study, EEG was used to investigate brain-related changes to oscillatory activity. The present study did not include EEG measures, and so it is not possible to conclusively assess the effect of the tACS on oscillatory brain activity. However, it is reasonable to expect that similar changes should be induced in motor regions compared to non-motor regions. We therefore speculate that the application of tACS in the present study might have increased local slow wave activity (and probably also theta activity), and that this contributed to the longer-term effects in the active condition compared to sham stimulation. However, it should also be noted that we did not specifically investigate the frequency or spatial specificity of the tACS applied in the present experiment. Indeed, research investigating other frequencies of tACS have also shown complimentary effects. For example, tACS applied at a beta frequency following a serial reaction time task improved reaction times during the retrieval period [32]. Also, high gamma (70 Hz) tACS has also been demonstrated to be effective in promoting motor learning when applied after a visually cued button press task [33]. Thus, tACS at various frequencies can play an important role in promoting processes associated with motor learning, but act on processes aligned with the targeted tACS frequency. Our research adds to this body of work, suggesting that slow oscillatory tACS, applied during the motor learning, can promote more longer-lasting changes in performance.

The second approach used to investigate the effects of motor training and tACS in the present study was with the use of TMS to quantify peak-to-peak MEP amplitude. The average MEPs in the active and sham groups did not significantly change following the motor learning task, or 24 h later, compared to baseline, nor were they affected by tACS. This is an unexpected result, as previous studies have suggested that motor learning alone without additional stimulation will result in a significant change in cortical MEPs [29]. A likely explanation for this finding is that we did not measure MEP changes for sufficient time post-training to allow the cortical changes arising from training to manifest. Previous research has shown that MEP changes arising from ballistic motor training are not present immediately after training, but are present 15 min after the completion of training [34]. Therefore, it may be that training did lead to MEP changes, but that we did not probe cortical excitability for a sufficiently long period. It is possible that factors which modulate corticospinal excitability such as emotion, genetics and amount of sleep may have affected outcomes [35]. Another potential explanation for the results is that the MEPs of the cortical representation of the APB were assessed during rest, whereas the tACS was applied during an active task. Thus the training and tACS may have caused state-dependent changes to the neuronal networks which were not effectively probed with resting-state TMS measures (for review see [36]). Therefore, in future, MEPs should be acquired during rest, and during a tonic voluntary contraction to take into account any state-dependent changes in brain activity.

This study investigated the effects of a single, 30 min motor training session with concurrent slow-wave oscillatory tACS during wakefulness. When receiving active stimulation, participants showed an increase in training-related change in thumb acceleration 24 h later. Thus, the longer-term consolidation of a motor training task benefits from the concurrent application of tACS during training. This has potential implications in the clinical sphere. Rehabilitation after stroke often requires repeated practice of motor tasks in order to improve functional outcomes. Sleep has been shown to be an important factor in improving rehabilitation in stroke patients [37]. Sleep plays a pivotal role in consolidating memories and skills that were acquired during the previous day [4, 38]. In particular, the periods of quiescence and bursts of large amplitude activity spikes which characterize NREM sleep are thought to play a crucial role in consolidating motor and memory function [38, 39]. In the present study, we show that one of the main features of NREM sleep – high amplitude, low frequency (~ 1 Hz) oscillations in brain activity—can assist in training-related improvements in motor learning even in the awake brain. We certainly acknowledge two limitations when considering the sleep-related aspects of the present study. First, we did not collect any sleep data (e.g., polysomnography, sleep diary) and so we are unable to determine if a) our participants slept normally following their initial experimental session, and b) whether there were differences in sleep quality/duration between groups. Second, NREM sleep also consists of other physiological characteristics, including sleep spindles and sharp wave ripples [40] that also play a role in plasticity effects, and have also been targeted effectively by tACS [17]. However, the present study shows that low frequency oscillations applied during wakefulness appear to mimic sleep-like processes by improving the retention of training-related changes in the awake brain. Given that tACS is portable, affordable, painless and safe, it offers a potential adjunct treatment in stroke rehabilitation. However, to further investigate the potential impact of tACS, it will be important to test its efficacy in an older population in future studies, as the brain’s response to non-invasive brain stimulation and potential for neuroplasticity is altered with age [11]. Future avenues of research should then explore practical uses of tACS combined with motor learning, such as retraining a functional task in a neurorehabilitation setting in patients following a stroke or traumatic brain injury. Numerous studies have suggested the high prevalence of impaired sleep in patients following haemorrhagic or ischaemic stroke due to factors such as insomnia and breathing disturbances [41] affecting as many as 78% of patients [42]. In another study, increased sleep time, sleep efficiency and NREM sleep phase were associated with better functional outcomes in patients following stroke [43]. If tACS can be applied to mimic the beneficial aspects of slow wave sleep, even during wakefulness, patients with impaired sleep may still gain benefit from motor learning. Therefore, the encoding effects of slow-wave oscillations are particularly important to investigate in this population due to its potential impact on the efficiency of motor learning, and subsequent functional recovery.

In summary, the application of slow-wave 0.75 Hz tACS applied concurrently with a motor training task significantly improved the consolidation of the motor learning task 24 h later compared to participants who received the sham stimulation. This finding was evident in changes in thumb acceleration – despite both groups showing an increase in acceleration 24 h after training, participants in the active stimulation group demonstrated a significantly faster mean horizontal thumb abduction acceleration compared to participants in the sham stimulation group at this time point. However, there were no TMS-evoked cortical changes following combined tACS and motor training. These results provide preliminary support for the efficacy of slow oscillatory tACS to enhance the effects of a simple motor training paradigm. This may have important implications for the use of tACS in a neurorehabilitation setting, especially following stroke and traumatic brain injury.

## Data Availability

The data that support the findings of this study are openly available in the Open Science Framework at https://osf.io/z7en2/#:~:text=DOI%2010.17605/OSF.IO/Z7EN2
